# Survival Outcomes of Local Compared With Systemic First Treatment of Non-Small Cell Lung Cancer Brain Metastases

**DOI:** 10.3389/fonc.2021.706409

**Published:** 2021-11-10

**Authors:** Hong-Mei Liu, Chun-Liu Meng, Lu-Jun Zhao

**Affiliations:** ^1^ Department of Radiation Oncology, Tianjin Medical University Cancer Institute and Hospital, National Clinical Research Center for Cancer; Key Laboratory of Cancer Prevention and Therapy, Tianjin; Tianjin’s Clinical Research Center for Cancer, Tianjin, China; ^2^ Department of Radiation Oncology, Tangshan Gongren Hospital, Tangshan, China

**Keywords:** brain metastases, non-small cell lung cancer, local treatment, systemic treatment, initiation of local treatment

## Abstract

**Objective:**

This retrospective study evaluated the survival advantage of local treatment targeted to brain metastases, relative to systemic therapy, as the first option for brain metastases of non-small cell lung cancer (NSCLC).

**Methods:**

First reviewed were 291 cases of NSCLC brain metastases from two centers. All patients were at least 18 years old, with histologically confirmed NSCLC, and required and underwent both local (radiotherapy or brain surgery) and systemic treatment (chemotherapy and tyrosine kinase inhibitor [TKI] medication). Demographics, clinical characteristics, and treatment-related variables were collected.

**Results:**

The final population comprised 160 patients. Overall, the multivariate analysis suggested that the following were associated with better survival: >3 cycles of chemotherapy; stereotactic radiosurgery; and TKI medication (all, P = 0.000). Local treatment that began within 1 week of the diagnosis of brain metastases was associated with poorer survival (P = 0.006). Among the 111 patients with symptomatic brain metastases, the multivariate analysis indicated that better survival was associated with >3 cycles of chemotherapy (P = 0.000), radiation dose >40 Gy (P = 0.001), stereotactic radiosurgery (P = 0.000), and TKI medication (P = 0.000), while local treatment that began within 1 week after the diagnosis of brain metastases was associated with poorer survival (P = 0.015).

**Conclusions:**

For patients with NSCLC brain metastases, regardless of the presence of clinical symptoms associated with brain metastases, systemic treatment before local may be better for survival. Even when used to relieve clinical symptoms, local treatment should be within a setting of sufficient systemic treatment.

## Introduction

Lung cancer is the deadliest malignancy in both China and the United States (1, 2). The incidence of lung cancer metastasized to the brain is about 30% to 50% and seriously threatens the life of patients (3). Left untreated, the median overall survival (OS) time of patients with lung cancer brain metastases is 3 to 6 months, or even less (4). With rapid developments in medicine, the median survival time has become about 16 months (5).

The treatment of lung cancer brain metastases involves both local (surgery and radiation) and systemic therapies (molecularly targeted agents, chemotherapy, and immunotherapy) (6, 7), depending on whether patients are symptomatic or asymptomatic. Chinese and American guidelines generally recommend that patients with symptomatic brain metastases receive both local and systemic treatment, especially for intracranial masses. Patients who are asymptomatic receive systemic treatment, and local treatment is applied as necessary (8, 9).

The present retrospective study addressed whether the treatment sequence, that is, whether systemic or local therapy is applied first, influences survival outcomes in both symptomatic and asymptomatic patients.

## Materials and Methods

### Data Collection

The records of 291 patients with brain metastases of non-small cell lung cancer (NSCLC) were reviewed at Tianjin Medical University Cancer Institute and Hospital (2015.1–2017.1) and Tangshan Gongren Hospital (2011.3–2019.1). Patients included in the analysis met the following criteria: at least 18 years of age; with histologically confirmed NSCLC; and requiring both local and systemic treatment. Among the initial 291 patients considered, 131 were excluded for receiving local treatment only, systemic treatment only, or neither treatment. Local treatment meant targeted to the brain metastases and included radiotherapy and brain surgery. Systemic treatment was directed toward all lesions, such as chemotherapy and tyrosine kinase inhibitor (TKI) medication. Examples of the latter are epidermal growth factor receptor (EGFR) TKI and anaplastic lymphoma kinase (ALK) TKI. The use of immune checkpoint inhibitors was not included in this analysis.

Finally, 160 patients met the above criteria. Data concerning background, clinical characteristics, and treatment-related variables were collected. The following stratification factors were selected according to clinical knowledge: age; Karnofsky Performance Scale (KPS) score; brain metastatic symptoms; brain metastatic lesions; extracranial metastases; primary lesion control; and liver metastases. Factors pertaining to treatment were type and starting time of initial treatment, the number of chemotherapy cycles, TKI medication, and the cycle of systemic treatment at which local treatment began, and radiation dosage.

### Follow-Up

Telephone follow-ups and search of the hospital medical record systems were conducted by C-LM at Tianjin Medical University Cancer Institute and Hospital and H-ML at Tangshan Gongren Hospital. If the patient’s phone was disconnected, the last discharge date was considered the date of loss of follow-up (21 patients). OS was defined as the time from the diagnosis of brain metastases to tumor-related death or loss of follow-up. The end date of the follow-up was 1 October 2020.

### Statistical Analysis

The Cox proportional hazard model was used for univariate and multivariate analyses. Chi-squared and Fisher’s exact test were used to analyze categorical variables. P < 0.05 was considered statistically significant. SPSS version 24.0 was used as the statistical software.

## Results

The study population comprised 160 patients (100 men, 60 women) who met the criteria and were treated from March 2011 to January 2019. Among them, there were 111 and 49 symptomatic and asymptomatic patients, respectively (**Table 1**). Local treatments for brain metastases were the following: three-dimensional conformal radiation therapy (3D-CRT), intensity-modulated radiation therapy (IMRT), stereotactic radiosurgery (SRS), or brain surgery. Among the 160 patients, 15 (9.4%) underwent brain surgery, and 52 (32.5%) were given TKI medication. A total of 50 patients took first-generation EGFR-TKI, and 1 patient each was administered, respectively, with second-generation EGFR-TKI and second-generation ALK-TKI. Nine patients were given osimertinib after progressive disease.

**Table 1:** Patient characteristics of the overall (n = 160) and symptomatic (n = 111) populations, n.

		Overall	Symptomatic			Overall	Symptomatic
Subjects, n		160	111	CT cycles	≤3	99	72
Age, years	<65	114	77		>3	61	39
	≥65	46	34	Radiation method	3D-CRT/IMRT	141	101
Gender	Male	100	71		SRS	19	10
	Female	60	40	Radiation dose	≤40 Gy	84	57
Smoking history	Yes	99	72		>40 Gy	76	54
	No	61	39	TKI medication	Yes	52	37
KPS score	<70	4	3		No	108	74
	≥70	156	108	Start LT, w	≤1	41	31
BM symptoms	Yes	111	49		>1	119	80
	No	49	Nil		≤2	90	71
BM lesions	Single	52	30		>2	70	40
	Multiple	108	81		≤3	115	85
Metastases, extracranial	Yes	81	53		>3	45	26
	No	79	58		≤4	119	89
Metastases, liver	Yes	17	10		>4	41	22
	No	143	104	Cycle of CT at LT start	<2	126	94
Primary lesion control	Yes	41	29		≥2	34	17
	No	119	82		<3	136	99
Initial treatment	Local	99	78		≥3	24	12
	Systemic	61	33		<4	143	103
					≥4	17	8

BM, brain metastasis; CT, chemotherapy; LT, local therapy.

**Figure 1:**
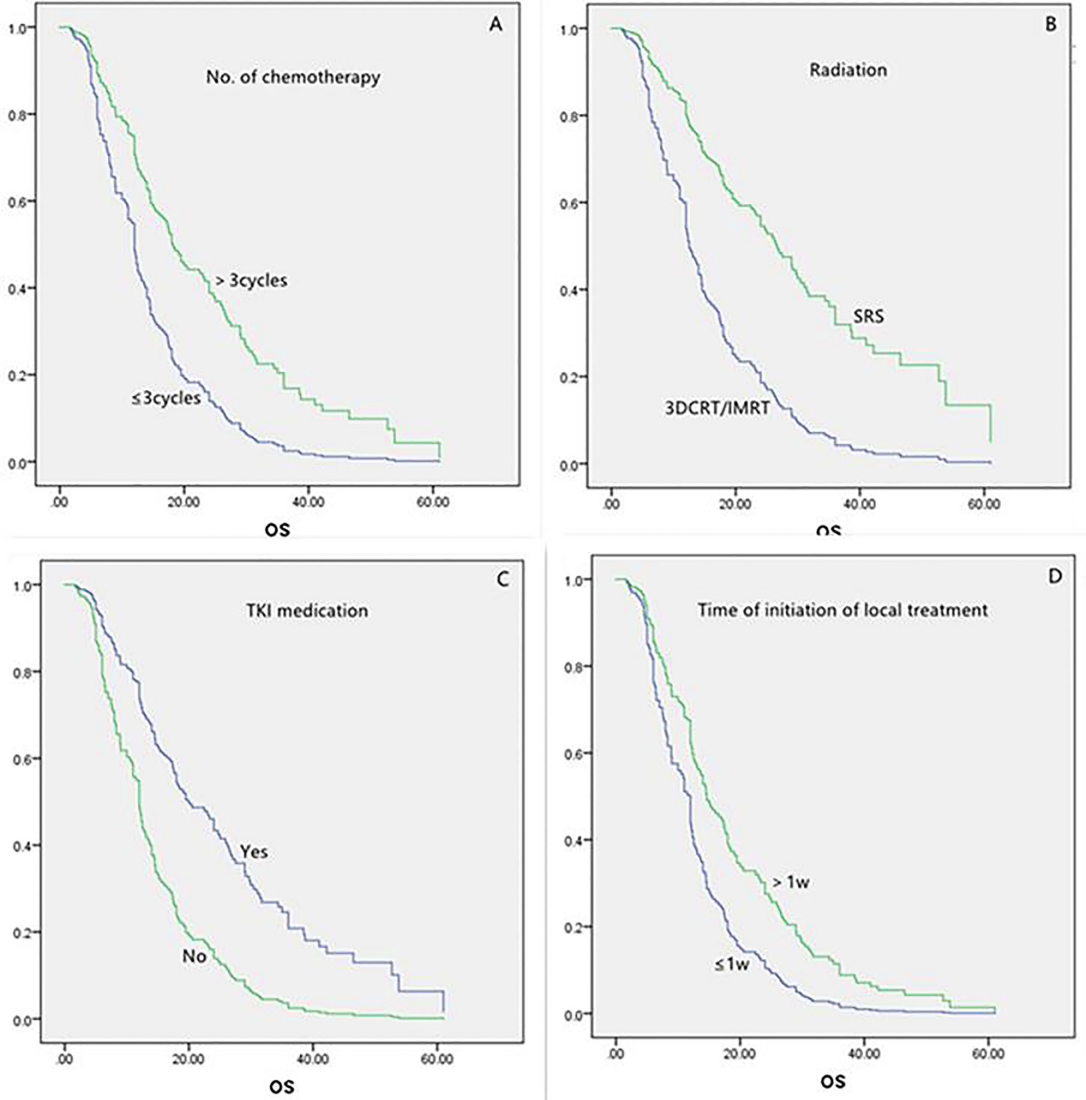
Multivariate analysis of variables in the overall populations.

For all the 160 patients, the univariate analysis showed that longer survival was linked to the following: age less than 65 years, more than three chemotherapy cycles, SRS, and TKI medication (**Table 2**). Local treatment proceeding systemic treatment was associated with shorter survival rather than *vice versa*. The multivariate analysis showed that longer survival was associated with more than three chemotherapy cycles, SRS, and TKI medication (each, P = 0.000; **Table 3** and **Figure 1**). The initiation of local treatment within 1 week of diagnosis of brain metastases was associated with shorter survival (P = 0.006).

**Table 2:** Univariate analysis of variables in overall (n = 160) and symptomatic (n = 111) populations, *P* values.

	Overall population	Symptomatic
Age	0.006 (HR=1.654,95%CI 1.154-2.372)	0.005 (HR=1.858,95%CI 1.205-2.865)
Gender	0.893	0.525
Smoking history	0.702	0.803
KPS score	0.803	0.518
BM symptoms	0.941	—
BM number	0.388	0.219
Metastases, extracranial	0.275	0.073
Metastases, liver	0.267	0.663
Primary lesion control	0.067	0.123
Initial treatment type	0.088	0.349
No. of CT cycles	0.004 (HR=1.655,95%CI 1.177-2.326)	0.017 (HR=1.683,95%CI 1.098-2.581)
Radiation method	0.001 (HR=2.440,95%CI 1.411-4.220)	0.018 (HR=2.451,95%CI 1.170-5.136)
Radiation dose	0.751	0.154
TKI medication	0.001 (HR=0.551,95%CI 0.382-0.794)	0.005 (HR=0.526,95%CI 0.337-0.820)
Week of LT start		
1	0.002 (HR=1.847,95%CI 1.248-2.732)	0.024 (HR=1.707,95%CI 1.072-2.719)
2	0.013 (HR=1.522,95%CI 1.093-2.119)	0.024 (HR=1.627,95%CI 1.067-2.482)
3	0.101	0.085
4	0.070	0.050 (HR=1.671,95%CI 0.999-2.792)
Cycle of CT at LT start		
2	0.184	0.469
3	0.276	0.644
4	0.096	0.377

**Table 3:** Multivariate analysis of variables in the overall (n = 160) and symptomatic (n = 111) populations.

Overall population			Symptomatic population		
	HR (95%CI)	*P*		HR (95%CI)	*P*
No. of CT cycles	2.084 (1.469-2.956)	0.000	Radiation	4.353 (1951-9.716)	0.000
Radiation	2.778 (1.578-4.889)	0.000	Dose	2.162 (1.395-3.352)	0.001
TKI medication	0.423 (0.291-0.616)	0.000	No. of CT cycles	2.507 (1.581-3.976)	0.000
Initiation of LT ≤1 w	1.754 (1.176-2.617)	0.006	TKI medication	0.314 (0.193-0.510)	0.000
—	—	—	Initiation of LT ≤1 w	1.832 (1.126-2, 979)	0.015

CT, chemotherapy; LT, local therapy.

**Figure 2:**
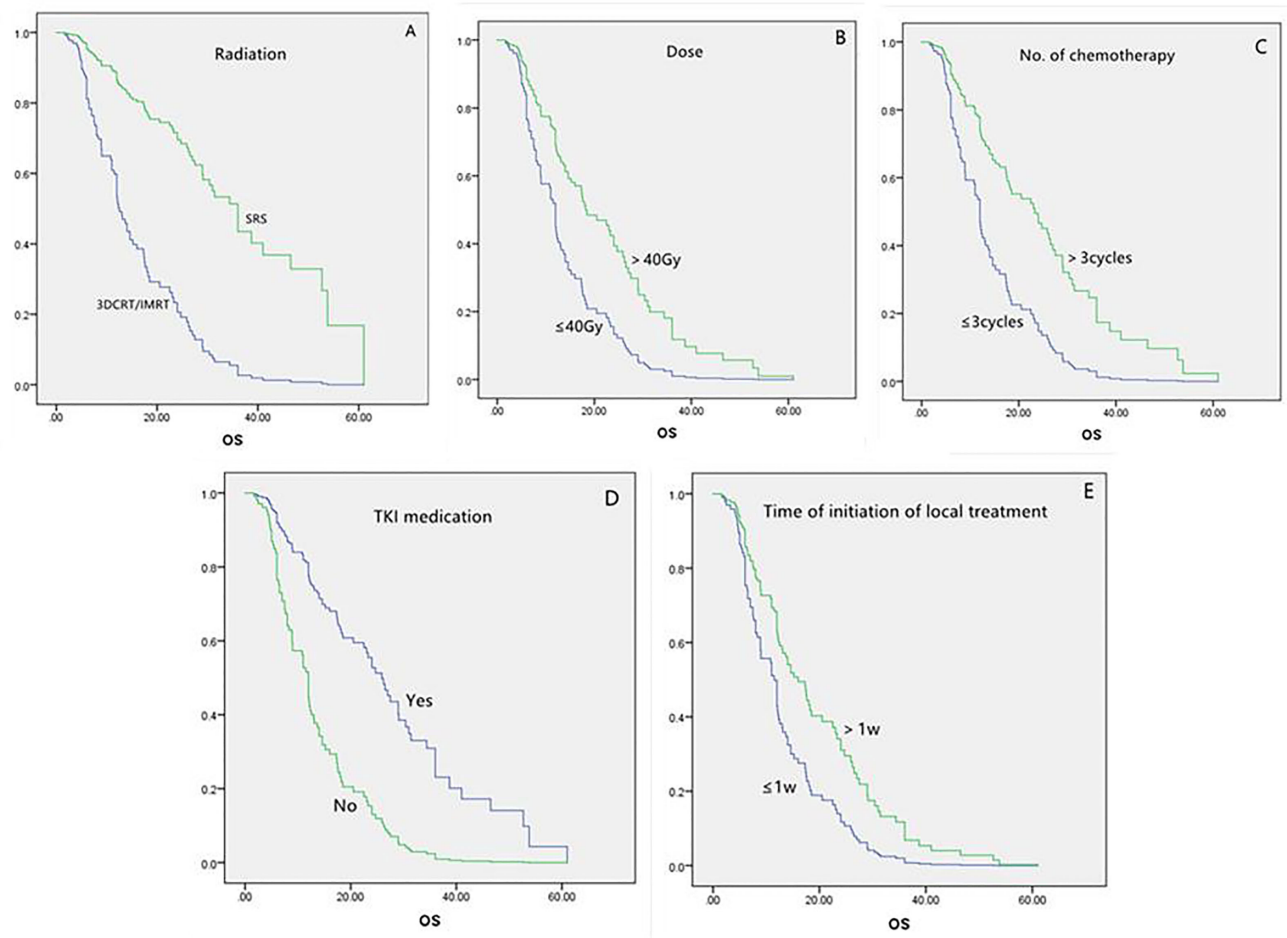
Multivariate analysis of variables in the symptomatic populations.

For the group of 111 patients with symptomatic brain metastases, the univariate analysis showed that factors that led to longer survival were the following: younger than 65 years, more than three chemotherapy cycles, SRS, and TKI medication (**Table 2**). Local treatment proceeding systemic treatment was associated with shorter survival rather than *vice versa*. Multivariate analysis showed that longer survival was associated with more than three chemotherapy cycles (P = 0.000), SRS (P = 0.000), radiation dose >40 Gy (P = 0.001), and TKI medication (P = 0.000). Local treatment that was started within 1 week of the diagnosis of brain metastases was associated with shorter survival (P = 0.015; **Table 3** and **Figure 2**).

The patients were further stratified based on whether local treatment was given within, or after, 1 week of diagnosis (i.e., ≤1 w cf. >1 w; **Table 4**). Among the 160 patients overall, compared with the group that received local treatment after 1 week, the percentage of patients older than 65 years was significantly higher (P = 0.037), and the rate of liver metastases was greater (P = 0.032), in the group that was given local treatment within 1 week. None of the patients in the latter group received SRS (P = 0.004). Considering only the 111 patients with symptomatic brain metastases, there were no significant differences in variables between the two groups.

**Table 4:** Patient characteristics of the overall and symptomatic populations after stratified.

		Overall population (n = 160)			Symptomatic(n = 111)		
		≤1 w	>1 w	*P*	≤1 w	>1 w	*P*
Age, y	>65	24	90	0.037	18	59	0.108
	≥65	17	29		13	21	
Gender	Male	27	73	0.607	19	52	0.715
	Female	14	46		12	28	
Smoking history	Yes	25	74	0.891	17	55	0.168
	No	16	45		14	25	
KPS score	>70	1	3	0.729	1	2	0.630
	≥70	40	116		30	78	
BM symptoms	Yes	31	80	0.315	31	80	—
	No	10	39		—	—	
BM number	Single	14	38	0.794	11	19	0.212
	Multiple	27	81		20	61	
Metastases, liver	Yes	8	9	0.032	5	5	0.103
	No	33	110		26	75	
Primary lesion control	No	27	92	0.147	19	63	0.060
	Yes	14	27		12	17	
CT cycles	≤3	26	73	0.814	18	54	0.350
	>3	15	46		13	26	
Radiation method	3DCRT/IMRT	41	100	0.004	31	70	0.059
	SRS	0	19		0	10	
Radiation dose, Gy	≤40 Gy	18	66	0.201	13	44	0.217
	>40 Gy	23	53		18	36	
TKI medication	Yes	13	39	0.900	10	27	0.881
	No	28	80		21	53	

BM, brain metastasis; CT, chemotherapy.

## Discussion

The results of this study suggest that local treatment within 1 week of diagnosis of NSCLC brain metastases, prior to systemic treatment, leads to a shorter survival.

Our results are consistent with some previous studies (10–12). In a phase III clinical trial, Robinet et al. (10) confirmed the efficacy of chemotherapy for treating brain metastases of NSCLC, and concurrent whole brain radiation therapy (WBRT), early or delayed, did not influence survival. Chen et al. (11) found that the median survival associated with combined systemic and local therapy was longer than that of either systemic or local therapy alone. In the same study, however, the median OS of patients given combined systemic and early brain (i.e., local) radiotherapy was inferior to that of patients given combined systemic therapy and deferred brain radiotherapy. Liu et al. (12) showed that, for patients with EGFR mutation and without symptoms of brain metastasis, first-line systemic treatment was associated with longer survival compared with first-line radiotherapy. The above three studies suggest that, for patients with brain metastases, systemic treatment prior to local treatment was more favorable for prognosis than *vice versa*.

To investigate the poorer prognosis of patients who undergo local treatment within 1 week of diagnosis, the present study compared the clinical features of the groups given early (≤1 w) or late (>1 w) local treatment. Remembering that this was a retrospective study and that patients were not randomly assigned to a specific treatment, the group who received early local treatment contained a higher percentage of elderly patients, a higher percentage of patients complicated by liver metastases, and none of the patients who received SRS. Among the 19 patients who received SRS (i.e., in the late local treatment group), only 1 patient had liver metastases. This may be because of the acknowledged importance to prognosis of systemic treatment for patients with liver metastases (13, 14). And this reminded us that systemic treatment must be given first for patients both with brain metastases and liver metastases.

In a phase III clinical trial (15) concerning the treatment sequence for asymptomatic cerebral oligo-metastases (1-4 lesions) in NSCLC, SRS and then chemotherapy did not improve OS, relative to upfront chemotherapy. Thus, for asymptomatic patients with brain metastases, systemic treatment is preferred first. However, there have been no comparable clinical studies concerning the sequence of treatment for patients with symptomatic brain metastases. The present study indicates that early local treatment (≤1 w) leads to a poor prognosis, and there was no significant difference in clinical features between early local treatment (≤1 w) and late local treatment(>1 w).Therefore, for the patients with symptomatic brain metastasis, systemic treatment first was more appropriate, and local treatment is added to relieve symptoms. If radiotherapy was given to the brain, our study showed that higher doses (>40 Gy) were better for prognosis. Meng et al. (16) also reported that patients who were treated with whole brain radiotherapy plus focal radiation boost experienced significantly longer OS and intracranial progression-free survival than did those with whole brain radiotherapy alone or SRS alone.

The present study also showed that sufficient systemic therapy and TKI medication were associated with longer survival. This has been demonstrated in previous studies (17, 18). Bo et al. (17) showed that systemic therapy plus either SRS or whole brain radiotherapy may significantly reduce the risk of mortality compared with radiotherapy alone for patients with one to three brain metastatic lesions of NSCLC. Hironori et al. (18) reported that, for patients with newly diagnosed brain metastases of NSCLC, the OS was significantly longer than that of patients without a driver mutation.

The present study is limited by the defects that are inherent to a retrospective study, including that the number of brain metastases count not be counted. It should also be noted that systemic therapy did not always include immunotherapy, and that adverse effects associated with brain radiotherapy were not considered.

## Conclusions

Our data showed that for patients with NSCLC brain metastasis, regardless of the clinical symptoms associated with brain metastasis, systemic treatment first may be better for survival. Even if local treatment is used to relieve the clinical symptoms, it should be conducted under the condition of sufficient systemic treatment.

## Data Availability Statement

The datasets presented in this study can be found in online repositories. The names of the repository/repositories and accession number(s) can be found in the article/supplementary material.

## Author Contributions

H-ML and C-LM had full access to all of the data in the study and take responsibility for the integrity of the data and the accuracy of the data analysis. L-JZ contributed to the study design. H-ML contributed to data acquisition and critical history review. All authors contributed to the article and approved the submitted version.

## Conflict of Interest

The authors declare that the research was conducted in the absence of any commercial or financial relationships that could be construed as a potential conflict of interest.

## Publisher’s Note

All claims expressed in this article are solely those of the authors and do not necessarily represent those of their affiliated organizations, or those of the publisher, the editors and the reviewers. Any product that may be evaluated in this article, or claim that may be made by its manufacturer, is not guaranteed or endorsed by the publisher.
